# Pigment epithelium-derived factor reduces apoptosis and pro-inflammatory cytokine gene expression in a murine model of focal retinal degeneration

**DOI:** 10.1042/AN20130028

**Published:** 2013-11-26

**Authors:** Yujuan Wang, Preeti Subramanian, Defen Shen, Jingsheng Tuo, S. Patricia Becerra, Chi-Chao Chan

**Affiliations:** *Immunopathology Section, Laboratory of Immunology, National Eye Institute, National Institutes of Health, Bethesda, MD 20892, U.S.A.; †Zhongshan Ophthalmic Center, Sun Yat-sen University, Guangzhou 510060, China; ‡Section of Protein Structure and Function, Laboratory of Retinal Cell and Molecular Biology, National Eye Institute, National Institutes of Health, Bethesda, MD 20892, U.S.A.

**Keywords:** age-related macular degeneration, apoptosis, inflammation, neuroprotection, pigment epithelium-derived factor, retina, A2E, [2,6-dimethyl-8-(2,6,6-trimethyl-1-cyclohexen-1-yl)-1E,3E,5E,7E-octatetra-enyl]-1-(2-hydroxyethyl)-4-[4-methyl-6(2,6,6-trimethyl-1-cyclohexen1-yl) 1E,3E,5E,7E-hexatrienyl]-pyridinium, AMD, age-related macular degeneration, CNS, central nervous system, CNV, choroidal neovascularization, Crb1, crumbs-like 1, DAPI, 4′,6-diamidino-2-phenylindole dihydrochloride, DKO *rd8*, *Ccl2^−/−^/Cx3cr1^−/−^* on C57BL/6N [*Crb1^rd8^*], DMEM/F12, Dulbecco’s modified Eagle’s medium: Ham’s nutrient mixture F-12, FasL, Fas ligand, IL-17a, interleukin-17a, IL-1β, interleukin-1 beta, INL, inner nuclear layer, iNos, inducible nitric oxide synthase, IS/OS, inner/outer segment, ONL, outer nuclear layer, OPL, outer plexiform layer, PEDF, pigment epithelium-derived factor, qRT-PCR, quantitative reverse transcription-PCR, rd, retinal degeneration, RPE, retinal pigment epithelium, TNFα, tumor necrosis factor-alpha, TUNEL, terminal deoxynucleotidyl transferase dUTP nick end labeling, VEGFA, vascular endothelial growth factor A, WT, wild-type

## Abstract

AMD (age-related macular degeneration) is a neurodegenerative disease causing irreversible central blindness in the elderly. Apoptosis and inflammation play important roles in AMD pathogenesis. PEDF (pigment epithelium-derived factor) is a potent neurotrophic and anti-inflammatory glycoprotein that protects the retinal neurons and photoreceptors against cell death caused by pathological insults. We studied the effects of PEDF on focal retinal lesions in DKO *rd8* (*Ccl2^−/−^/Cx3cr1^−/−^* on C57BL/6N [*Crb1^rd8^*]) mice, a model for progressive, focal rd (retinal degeneration). First, we found a significant decrease in PEDF transcript expression in DKO *rd8* mouse retina and RPE (retinal pigment epithelium) than WT (wild-type, C57BL/6N). Next, cultured DKO *rd8* RPE cells secreted lower levels of PEDF protein in the media than WT. Then the right eyes of DKO *rd8* mice were injected intravitreously with recombinant human PEDF protein (1 μg), followed by a subconjunctival injection of PEDF (3 μg) 4 weeks later. The untreated left eyes served as controls. The effect of PEDF was assessed by fundoscopy, ocular histopathology and A2E {[2,6-dimethyl-8-(2,6,6-trimethyl-1-cyclohexen-1-yl)-1E,3E,5E,7E-octatetra-enyl]-1-(2-hydroxyethyl)-4-[4-methyl-6(2,6,6-trimethyl-1-cyclohexen-1-yl) 1E,3E,5E,7E-hexatrienyl]-pyridinium} levels, as well as apoptotic and inflammatory molecules. The PEDF-treated eyes showed slower progression or attenuation of the focal retinal lesions, fewer and/or smaller photoreceptor and RPE degeneration, and significantly lower A2E, relative to the untreated eyes. In addition, lower expression of apoptotic and inflammatory molecules were detected in the PEDF-treated than untreated eyes. Our results establish that PEDF potently stabilizes photoreceptor degeneration via suppression of both apoptotic and inflammatory pathways. The multiple beneficial effects of PEDF represent a novel approach for potential AMD treatment.

## Abstract 2

Apoptosis and inflammation play important roles in age-related macular degeneration. As a potent neurotrophic and anti-inflammatory glycoprotein, PEDF potently stabilizes photoreceptor degeneration via suppression of apoptotic and inflammatory pathways in a mouse model of progressive, focal rd.

## INTRODUCTION

AMD (age-related macular degeneration) is a chronic, progressive, neurodegenerative disorder in the retinal macula. AMD has become the leading cause of irreversible central vision loss in the elderly worldwide (Coleman et al., 2008; Klein et al., 2011; Lim et al., 2012). There are two major forms of AMD: geographic atrophic (dry) AMD and exudative/neovascular (wet) AMD. Atrophic AMD is characterized by drusen deposits, RPE (retinal pigment epithelium)/photoreceptor degeneration and loss in the macula. In neovascular AMD, CNV (choroidal neovascularization) breaks through the Bruch's membrane to the neuroretina, causing fluid leakage, lipid accumulation, and ultimately resulting in fibrovascular scarring. Most AMD patients have large confluent drusen in the macula and later develop geographic atrophy or neovascular AMD, both of which cause severe visual loss (Ambati and Fowler, 2012).

Although AMD is a neurodegenerative disorder (de Jong, 2006), causes of the macular degeneration are still not clear. Several evidence has linked it to intense parainflammation due to a wide range of causes, such as ageing, oxidative stress, genetic predisposition, and environmental elements (Xu et al., 2009; Rutar et al., 2011; Wang et al., 2011; Ambati and Fowler, 2012; Colak et al., 2012; Kauppinen et al., 2012; Tarallo et al., 2012). Under these conditions, the RPE, photoreceptors and retinal neurons are exposed to the cytokines and neurotoxins released by the activated inflammatory components, thereby contributing to the initiation and progression of AMD. In addition, studies have shown that apoptosis of RPE and photoreceptors are involved in geographic atrophy AMD (Dunaief et al., 2002; Yang et al., 2005).

PEDF (pigment epithelium-derived factor) is a naturally occurring glycoprotein of 50-kDa that is present abundantly in the retina and is secreted mainly by RPE in a directional fashion toward the neuroretina (Becerra et al., 2004). It belongs to the serpin (serine protease inhibitor) superfamily by sequence homology (Steele et al., 1993). Although PEDF lacks protease inhibitory activity, it exhibits several biological activities of interest for the eye that make it a potential therapeutic agent for several ocular diseases. PEDF was discovered in the culturing media of human fetal RPE cells with potent neuronal differentiating activity on retinoblastoma cells (Tombran-Tink et al., 1991). Later it was established that PEDF is a neurotrophic factor of the retina and the CNS (central nervous system). It protects photoreceptors against apoptosis in the rd (retinal degeneration) and rds (retinal degeneration slow) and light damage animal models, inner retina against ischemia and retinal ganglion cells (Cayouette et al., 1999; Cao et al., 2001; Takita et al., 2003; Pang et al., 2007). PEDF has pro-survival and anti-apoptotic activity on R28 cells, a rat retinal progenitor cells in culture (Murakami et al., 2008; Fitzgerald et al., 2012). In a dose-dependent manner, PEDF effectively protects R28 cell death induced by serum starvation and elevates the levels of the anti-apoptotic Bcl2 gene and protein. Furthermore, it not only supports neural survival and neurite outgrowth in the retina and CNS but also has anti-inflammatory activity in Müller and endothelial cells (Marciniak et al., 2006; Yabe et al., 2010; Miyazaki et al., 2011; Park et al., 2011; Liu et al., 2012). In 1999, Dawson et al. reported that PEDF is one of the more potent antiangiogenic factors, which has demonstrable inhibitory activity against ocular neovascularization *in vivo* (Dawson et al., 1999).

Given the reported neurotrophic, retina-protective and anti-inflammatory effects of PEDF in the retina, the aim of this study was to evaluate the neuroprotective and anti-inflammatory effects of PEDF on the retinal lesions in a DKO *rd8* (*Ccl2^−/−^/Cx3cr1^−/−^* on C57BL/6N [*Crb1^rd8^*]) mouse model of progressive, focal rd, mimicking certain features of human atrophic AMD (Chu et al., 2012).

## MATERIALS AND METHODS

### PEDF protein

Highly purified recombinant human PEDF was obtained from the culture media of BHK (baby-hamster kidney) cells harboring an expression plasmid containing full-length PEDF cDNA (Stratikos et al., 1996), by ammonium sulphate precipitation and cation- and anion-exchange column chromatography as described previously (Subramanian et al., 2012).

### Animals and drug treatment

We generated the DKO *rd8* mouse as a model of progressive, focal rd (Tuo et al., 2007; Chan et al., 2008). In addition to the *Ccl2^−/−^*/*Cx3cr1^−/−^* double knockout, the C57BL/6N mouse line has the *Crb1* (crumbs-like 1) mutation in homozygous form (Mattapallil et al., 2012). The DKO *rd8* mouse has earlier onset and higher penetrance than *Ccl2* and *Cx3cr1* single knockout strains (Chu et al., 2012; Chen et al., 2013). Although it is not a perfect model for AMD (Chu et al., 2012; Mattapallil et al., 2012), these mice develop certain features resembling human AMD lesions (Chu et al., 2012). The DKO *rd8* mice and age-matched WT (C57BL/6N [*Crb1^rd8^*]) mice were bred in-house. All animal experiments were performed under protocols approved by the National Eye Institute's Institutional Animal Care and Use Committee and were in compliance with the Association for Research in Vision and Ophthalmology Statement for the Use of Animals in Ophthalmic and Vision Research. The right eyes of 6-week-old DKO *rd8* mice were intravitreously injected with recombinant human PEDF protein (1 μg), followed by a second subconjunctival injection of PEDF (3 μg) 4 weeks later. Left eyes were untreated and served as controls.

### Isolation and culture of primary mouse RPE cells

Mouse RPE was isolated from retinas of WT and DKO *rd8* mice of 6–8 weeks old. Briefly, experimental animals were euthanized and their eyes were enucleated. The globes were dissected free of periocular connective tissue, transferred into 2% Dispase II (neutral protease, grade II, Roche) in PBS, and incubated at 37°C for 40–45 min. Dispase II activity was terminated by washing the globes three times in DMEM/F12 (Dulbecco's modified Eagle's medium: Ham's nutrient mixture F-12) medium plus 15% (v/v) FBS. The anterior segment was removed and the retina was dissected free from the underlying RPE-choroidal eyecups. The loosely adherent RPE cell layer was gently separated from the choroid and transferred to a 15-ml tube containing DMEM/F12, 15% (v/v) FBS and 1% l-glutamine–penicillin–streptomycin. The RPE suspension was planted on 24-well cell culture plates at 5% (v/v) CO_2_/37°C. The medium was changed after 5–6 days, and every 2–3 days thereafter. The RPE cells grew to form a confluent cell layer by 10 days and then incubated in the serum-free culture medium for 72 h. The culture medium was collected to measure PEDF protein level by ELISA/Western blot and the cells in the wells were used for RNA/total protein isolation.

### RNA isolation and qRT-PCR (quantitative reverse transcription-PCR)

Total RNA was isolated from primary mouse RPE cells and retina using an RNeasy Mini Kit (Qiagen). One μg RNA was reverse transcribed with Superscript II RNase H Reverse Transcriptase (Invitrogen) to 20 μl cDNA except for the determination of PEDF transcript. qRT-PCR was performed on the resulting cDNA using Brilliant SYBR Green QPCR Master Mix (Stratagene). The comparative cycle threshold value method, representing log transformation, was used to establish relative quantification of the fold changes in gene expression using ABI 7500 System (Applied Biosystems). Each 25 μl reaction volume contained 2×PCR master mix (SYBR Green/Rox; SABiosciences), 0.4 μM of each primer and 1 μl cDNA. The cDNA was amplified with specific primers for 40 cycles. *β-actin* (sense primer 5′-TCCCCCAACTTGAGATGTATGAAG-3′ and antisense primer 5′-AACTGGTCTCAAGTCAGTGTACAGG-3′) was used as a housekeeping gene for internal control. Primers of *β-actin*, *Fasl* (*Fas ligand*), *Fas*, *Bax*, *Bcl2*, *TNFα* (*tumor necrosis factor-alpha*), *Il1β* (*interleukin-1beta*), *Il17a* (*interleukin-17a*), *iNos* (*inducible nitric oxide synthase*) and *Vegfa* (*vascular endothelial growth factor A*) were purchased from SABiosciences. For PEDF, 200–400 ng mRNA was used for reverse transcription using SuperScript III First-Strand Synthesis System (Invitrogen). PEDF mRNA levels were quantified and normalized to 18S levels by quantitative RT-PCR using SYBR Green mix (Applied Biosystems) in the Bio-Rad Chromo4 real-time system. The thermal cycling conditions were 95°C for 15 min, then 46 cycles of 95°C for 30 s, 60°C for 30 s and 72°C for 30 s. The primers used were as follows: mouse PEDF 5′-ACCGTGACCCAGAACTTGAC-3′ (forward) and 5-CACGGGTTTGCCAGTAATCT-3′(reverse) and 18S 5′-GGTTGATCCTGCCAGTAG-3′ (forward) and 5′-GCGACCAAAGGAACCATAAC-3′(reverse).

### ELISA

PEDF protein levels in the culture medium were quantified by ELISA using the mouse PEDF ELISA kit (BioProducts MD, LLC). The media was treated with 8 M urea prior to ELISA as it resulted in better quantitation of PEDF levels. The samples were analyzed in duplicates following manufacturer's instruction. Standard curves of recombinant mouse PEDF were used to determine the sample concentration for each protein and normalized against the total protein concentration for each sample.

### Western blot

Different amounts of recombinant PEDF were also loaded along with the samples to estimate the amount of PEDF in the media upon densitometry analysis. Proteins were separated by SDS–PAGE using NuPAGE 10% PAGE in Bis–Tris buffer with NuPAGE MOPS–SDS as running buffer (Invitrogen) under reducing conditions. After separation by SDS–PAGE electrophoresis, proteins were transferred to a nitrocellulose membrane, blocked with blocking solution [1% (w/v) BSA in TBS-T (0.05 M Tris, pH 7.5, 0.05 M NaCl+0.1% (v/v) Tween 20)] for 1 h at room temperature (20–22°C), and incubated with polyclonal antibody to PEDF (Bioproducts MD, Inc.) in blocking solution at 1:10000 dilution and then washed three times with TBS-T for 5 min each. This was followed by incubations with secondary antibody (HRP-conjugated goat anti-rabbit IgG (KPL) diluted 1:200000 in blocking solution). For immunodetection, Super Signal West Dura Extended Duration Substrate (Thermo Scientific) was used and then the membrane was exposed to an X-ray film to visualize chemiluminescence signal.

### Fundus photography and clinical grading

Fundoscopy was performed before injection and 2 months post-injection. An endoscope with parallel illumination and observation channels was connected to a Nikon D90 digital camera. Mice were given intraperitoneal injection of ketamine (1.4 mg/mouse) and xylazine (0.12 mg/mouse) for systemic anesthesia and topical 1% tropicamide ophthalmic solution (Alcon Inc.) for pupil dilation. We assigned lesion grades by comparing the same fundus area over the 2-month course. Progression was defined as >10% increase in the number of the retinal lesions (+1), >50% increase in the lesion size in at least one-third of the lesions (+2), >5 fused lesions or the appearance of >2 chorioretinal scars (+3) and diffuse chorioretinal scars (+4). Regression was defined as >10% decrease in the number of retinal lesions (−1), >50% decrease in lesion size in at least one-third of the lesions (−2), >50% disappearance of retinal lesions (−3) and the total disappearance of retinal lesions (−4) (Tuo et al., 2012c). A masked observer conducted grading.

### A2E {[2,6-dimethyl-8-(2,6,6-trimethyl-1-cyclohexen-1-yl)-1E,3E,5E,7E-octatetra-enyl]-1-(2-hydroxyethyl)-4-[4-methyl-6(2,6,6-trimethyl-1-cyclohexen-1-yl) 1E,3E,5E,7E-hexatrienyl]-pyridinium} extraction and quantification

A2E is a lipofuscin fluorophore generated from the visual cycle flux of all-*trans*-retinol and is relevant to ageing and AMD pathogenesis (Ben-Shabat et al., 2001). Whole eyes were removed in a dark room under dim red light and homogenized. A2E was extracted with chloroform/methanol as previously described (Karan et al., 2005). The extracts dissolved in methanol were separated by HPLC (Agilent 1100 LC) and detected by an ultraviolet detector at a wavelength of 435 nm. A gradient of 40–95% (v/v) acetonitrile/H_2_O in 0.1 (v/v)% trifluoracetic acid was used to elute A2E on a reverse-phase C18 column (Agilent, eclipse XD8-C18, 5 μm, 4.6×150 mm) at a flow-rate of 1.0 ml/min. A2E was quantified using external A2E standards (Parish et al., 1998).

### Histopathology

Eyes were fixed for 30 min in 4% (v/v) gluteraldehyde followed by 10% (v/v) formalin for at least 24 h. Fixed eyes were embedded in methacrylate and serially sectioned in the vertical pupillary optic nerve plane. Each eye was cut into four sections and stained with hematoxylin and eosin, then analyzed under a light microscope.

### Frozen sections, TUNEL (terminal deoxynucleotidyl transferase dUTP nick end labeling) assay and immunohistochemistry

Eyes were harvested following euthanasia of mice, snap frozen and embedded in Tissue-Tek OCT Compound (Sakura Finetek USA Inc.). Serial sections were cut along the vertical pupillary-optic nerve plane and stored on slides at −70°C.

Frozen mouse eye slides were fixed in 4% (v/v) paraformaldehyde for 15 min, washed in PBS. The fixed slides were labeled using a TUNEL assay kit according to the manufacturer's instructions (Roche). In addition, the fixed slides were blocked in ICC buffer with 5% normal goat or rabbit serum for 30 min at 4°C. Samples were incubated overnight with primary antibodies to the following antigens: FasL, 1:50 (Abcam, Inc.); Fas, 1:200 (Santa Cruz); Bax, 1:200 (Santa Cruz); and Bcl-2, 1:200 (Santa Cruz). After washing with ICC buffer, DAPI (4′,6-diamidino-2-phenylindole dihydrochloride, 1:1000, Invitrogen) and secondary antibodies conjugated to either Alexa-488 or -555 (1:400, Invitrogen) were added and incubated at room temperature for 1 h. All the slides with TUNEL or immunohistochemical staining were examined under an Olympus FV1000 Confocal Scanning Microscope. TUNEL positive cells in the ONL (outer nuclear layer) were counted using the particles analyze plugin of the Image-J software. The thickness of ONL was measured by Olympus FV1000 software.

### Statistical analysis

Statistical analyses were performed using GraphPad Prism 5 (GraphPad Software). Cell data were evaluated using the unpaired *t* test. Mouse data of clinical fundus scores, qRT-PCR and A2E were compared using paired *t* test. All the probability levels in the paired and unpaired *t* test are two-tailed. A *P* value <0.05 was considered statistically significant.

## RESULTS

### Decreased PEDF expression in RPE and neuroretina of DKO *rd8* mice

**Figure 1:**
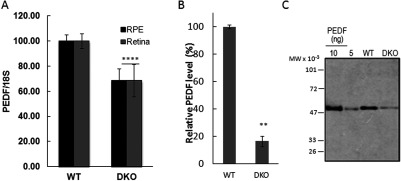
PEDF levels in RPE and retina of DKO *rd8* and WT (**A**) *Pedf* transcripts in freshly isolated retina and RPE from WT and DKO *rd8* mice by RT-PCR. *Pedf* transcripts are expressed relative to the expression of 18S (PEDF/18S ratio) and normalized to WT mice as 100%. Data are the average of three independent experiments. For RPE (*n*=16 and 4) and for retina (*n*=4 and 2) eyes were pooled in respective experiments. (**B**) PEDF protein concentration in the conditioned media of primary mouse RPE in culture was measured by ELISA. Each value corresponds to the average of duplicates per condition and with two mice per condition. Average of two individual experiments shows 84% of decrease in PEDF in DKO *rd8* mice compared with WT. (**C**) Western of conditioned media from RPE of DKO and WT against anti-PEDF (15 μl per lane). Standard PEDF amounts are for human recombinant PEDF 10 and 5 ng shown from left followed by WT and DKO. The migration position of molecular weight markers is given to the left (MW × 10^−3^).*indicates significant difference between DKO and WT as follows: **P<0.01; ****P<0.0001.

We compared the level of *Pedf* transcripts in freshly isolated RPE and neuroretina from WT and DKO *rd8* mice. *Pedf* was expressed in the retina of both WT and DKO *rd8.* Quantitation of expression level by real-time PCR revealed approximately 30% decrease in *Pedf* mRNA in DKO *rd8* RPE and neuroretina compared with WT (Figure 1A). Given that RPE is the main source for the secreted PEDF in the interphotoreceptor matrix (Becerra et al., 2004), primary mouse RPE cells were cultured from WT and DKO *rd8*, and the concentration of the secreted PEDF protein was measured in the serum-free conditioned media collected from these cells. The concentration of PEDF in DKO was 3-fold less than in WT (Figure 1B). The secreted PEDF protein in the conditioned media was analyzed by Western blot. The protein band migrated as expected for a 50 kDa size of PEDF protein and only one band was detected (Figure 1C). The estimated concentration of PEDF was 173 ng/ml for DKO and 650 ng/ml for WT, corresponding to a 3.7-fold decrease in the DKO mouse RPE, which is similar to that found by ELISA (Figure 1C). Overall, our data show a marked decrease in both PEDF mRNA and protein levels in DKO *rd8* mice compared with WT.

### PEDF attenuated focal retinal lesions of DKO *rd8* mice

**Figure 2:**
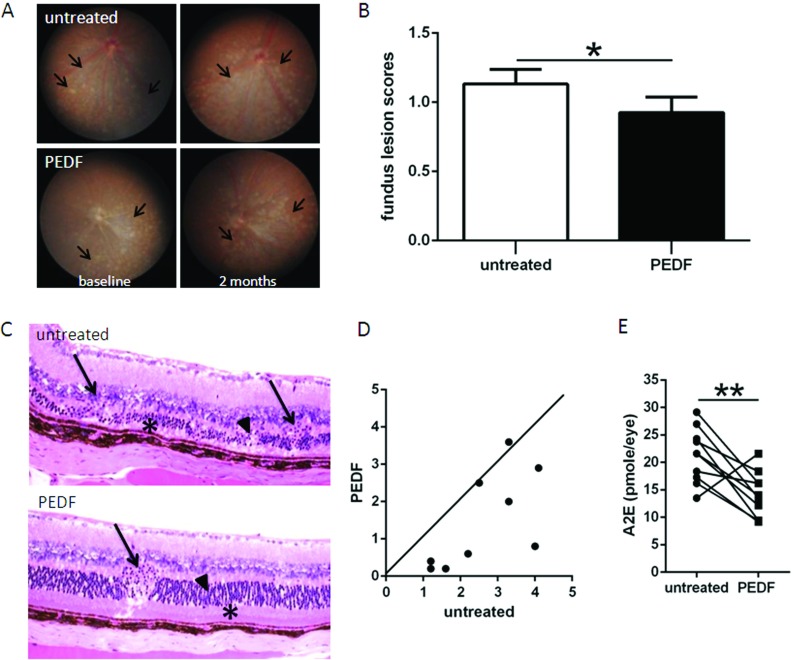
Retina-protective effect of PEDF in DKO *rd8* mouse retina (**A**) The fundus of PEDF-treated and untreated DKO *rd8* mouse eyes was taken by a Nikon D90 digital camera illuminated with an endoscope. Representative fundus findings showed changes of yellowish deep retinal lesions (arrows marked the margin of these lesions) in both eyes of the same mouse before and 2 months after PEDF treatment. (**B**) Fundus scores of 36 pairs of eyes based on the fundus pictures showing different progressing scores between the two eyes at the end of 2 months after PEDF treatment. (**C**) PEDF-treated and untreated DKO *rd8* mouse eyes were enucleated and embedded in methacrylate. Representative histopathological findings showed retinal lesions (shortening IS/OS and degenerative photoreceptors in the ONL, as well as dystrophy in the OPL) of both DKO and *rd8* associated lesions (arrows, OPL; asterisks, IS/OS; arrowheads, ONL). (**D**) Pairwise plotting of ten pairs of eyes showing different progressing scores between the two eyes. The dots below the line showed fewer lesions with PEDF treatment (*n*=8); the dot on the line showed similar pathological changes between two eyes (*n*=1); and the dot above the line showed worse lesions with PEDF treatment (*n*=1). (**E**) PEDF-treated and untreated DKO *rd8* mouse eyes were enucleated and homogenized for A2E extraction with chloroform/methanol (*n*=10).*indicates significant difference between untreated and PEDF-treated eyes as follows: *P<0.05; **P<0.01.

The decreased endogenous PEDF levels associated with the defects in the DKO eyes suggest that administration of PEDF could compensate such defects. To test whether PEDF would positively affect the retinal lesions in the DKO mice, we locally administered purified human recombinant PEDF protein. Fundoscopy revealed that PEDF-treated retina at 2 months showed significant improvement over the contralateral, untreated eyes, with fewer and smaller deep retinal lesions (Figure 2A, between arrows and 2B). Histopathologically, the untreated retina showed focal photoreceptor degeneration (Figure 2C, arrowheads), focal loss of IS/OS (inner/outer segment of photoreceptor, Figure 2C, asterisk), and RPE vacuolation/pigmentary alteration in addition to the *rd8* associated photoreceptor dystrophic lesions in OPL (outer plexiform layer, Figure 2C, arrows), whereas PEDF-treated retina presented fewer/smaller photoreceptor damages (Figure 2C, arrowhead) and relatively healthy photoreceptor IS/OS (Figure 2C, asterisk), normal RPE, but remaining *rd8* associated focal photoreceptor dystrophic lesions (Figure 2C, arrow). A total of ten pairs of eyes were histologically examined, eight mice had less lesions in PEDF-treated eyes compared with the contralateral, untreated eyes, one mouse had more lesions with PEDF treatment, and one mouse had similar pathological changes between the two eyes (Figure 2D). Moreover, PEDF treatment significantly reduced the levels of lipofuscin fluorophore A2E (Figure 2E).

### PEDF has anti-apoptotic effects on neuroretina of DKO *rd8* mice

**Figure 3:**
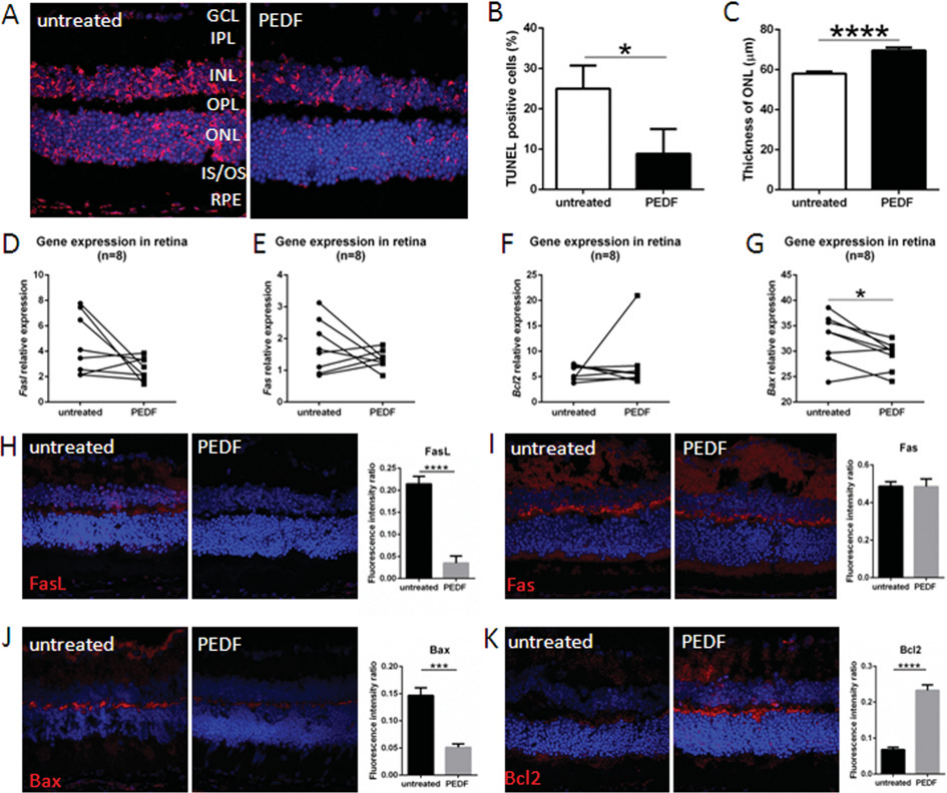
Anti-apoptotic effects of PEDF on the neuroretina in DKO *rd8* mice (**A**) The frozen sections of PEDF-treated and untreated DKO *rd8* eyes were stained with TUNEL assay (red color, *n*=5). (**B**) The percentage of TUNEL positive cells per total cells in the ONL is calculated by Image-J software (*n*=5). (**C**) The thickness of ONL of retina is measured by Olympus FV1000 software (*n*=5). Whole retina of PEDF-treated and untreated DKO *rd8* eyes was harvested and total RNA was isolated and converted to cDNA. To determine the transcript levels of targeted gene, the mRNA levels were quantified and normalized to universal mRNA with the *β-actin* housekeeping gene by qRT-PCR. (**D**) *Fasl*, (**E**) *Fas*, (**F**) *Bcl2* and (**G**) *Bax* (*n*=8). The frozen sections of PEDF-treated and untreated DKO *rd8* eyes were stained with apoptosis-related antibodies and immunoreactivity (red color) were evaluated under an Olympus FV1000 Confocal Scanning Microscope. Image-J software is used to measure the fluorescence intensity in pixels per area in each image and expressed as fluorescence intensity ratio. (**H**) anti-FasL, (**I**) anti-Fas, (**J**) anti-Bax and (**K**) anti-Bcl-2. (*n*=5). The nuclei were stained with DAPI (blue).*indicates significant difference between untreated and PEDF-treated eyes as follows: *P<0.05; ***P<0.001; ****P<0.0001.

As a useful tool to detect DNA fragmentation of apoptotic cells, TUNEL assay showed less apoptotic cells in PEDF-treated than contralateral, untreated retinas, especially in the ONL (Figures 3A and 3B). The thickness of ONL in the PEDF-treated eyes is significantly thicker than that in contralateral, untreated eyes (Figure 3C). Several apoptosis-related molecules were measured to investigate whether PEDF could attenuate retinal lesions via apoptotic pathways. Between PEDF-treated and contralateral, untreated eyes, *Fasl* (Figure 3D), *Fas* (Figure 3E) and *Bcl2* (Figure 3F) transcripts did not have significant differences in PEDF-treated than the contralateral, untreated retina, while the levels of *Bax* (Figure 3G) transcript were significantly decreased in PEDF-treated retina. Immunohistochemistry showed less immunoreactivity against FasL in the INL (inner nuclear layer) in PEDF-treated retina (Figure 3H) and comparable Fas in the INL, OPL and IS/OS in PEDF-treated and the contralateral, untreated retina (Figure 3I). In addition, lower Bax in the ganglion cell layer, OPL and IS/OS (Figure 3J) and higher Bcl-2 in the INL and OPL were observed in PEDF-treated than the contralateral, untreated retina (Figure 3K).

### PEDF inhibits angiogenic and inflammatory molecules on neuroretina of DKO *rd8* mice

**Figure 4:**
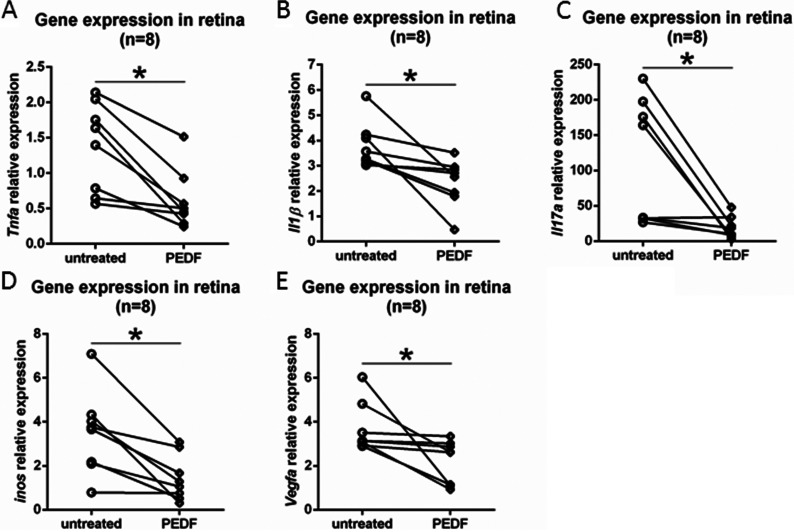
Anti-inflammatory and anti-angiogenic effects of PEDF on the neuroretina in DKO mice Whole retina of PEDF-treated and untreated DKO *rd8* eyes was harvested and total RNA was isolated and converted to cDNA. To determine the transcript levels of targeted gene, the mRNA levels were quantified and normalized to universal mRNA with the *β-actin* housekeeping gene by qRT-PCR. (**A**) *Tnfα*, (**B**) *Il1β*, (**C**) *Il17a*, (**D**) *iNos* and (**E**) *Vegfa* (*n*=8).*indicates significant difference between untreated and PEDF-treated eyes as *P<0.05.

There was significantly reduced expression of *Tnfα*, *Il1β*, *Il17a*, *iNos* and *Vegfa* transcripts in PEDF-treated than the contralateral, untreated retina (Figures 4A–4E).

## DISCUSSION

This study demonstrates that the PEDF level is markedly decreased in the retinas of DKO *rd8* mice with AMD-like lesions when compared with WT. Local administration of full-length PEDF ameliorates focal photoreceptor and RPE degeneration in this mouse model. More importantly, the neuroprotective effect of PEDF is associated with both anti-apoptotic and anti-inflammatory activities on retinal neuronal cells. PEDF-treated eyes showed fewer clinical and histopathological lesions in the retina, with less retinal cell apoptosis and better morphology in the ONL. Molecular studies demonstrated decreased apoptosis and inflammation with exogenous PEDF compared with the contralateral, untreated eyes. These results are in line with previous evidence that PEDF has neurotrophic and anti-inflammatory effects (Cayouette et al., 1999; Cao et al., 2001; Zhang et al., 2006; Pang et al., 2007; Zhou et al., 2009).

PEDF is widely expressed throughout the human body and displays multiple biological activities. Inside the eye, it is found as an extracellular component of the retinal interphotoreceptor matrix (Wu et al., 1995; Becerra et al., 2004). Given the neurotrophic and anti-inflammatory effects of PEDF, it is insinuated that decreased PEDF level can lead to neuroretinal degeneration and/or retinal cell apoptosis. As a focal rd mouse model with certain AMD features, RPE vacuolation and degeneration also occur in DKO *rd8* mice (Tuo et al., 2007; Chan et al., 2008). Because RPE is a main cell source producing PEDF in the eye, RPE abnormality could be responsible for decreased PEDF expression in the retinas of the DKO *rd8* mouse. This observation implies that PEDF deficiency may be one of the contributing factors causing focal rd in this AMD mouse model, which is in agreement with lower PEDF levels found in human RPE and choroid of AMD patients (Bhutto et al., 2006). We propose that local administration of PEDF may serve to restore PEDF levels inside the eye, exerting protective effects for the neuroretina in the DKO *rd8* mice, as well as a potential therapeutic agent for AMD patients.

RPE is intimately associated with photoreceptors by delivering essential nutrients for photoreceptors and phagocytizing shed photoreceptor outer segments. With ageing, non-phagocytic material accumulates in RPE as an increase of lipofuscin (Ben-Shabat et al., 2001). The major component of lipofuscin A2E causes further RPE damage and is regarded as a biomarker in AMD (Ben-Shabat et al., 2001; Wolf, 2003). It is impressive that not only PEDF treatment effectively reduced A2E level in the RPE of DKO *rd8* mice, but also improved the structural integrity of the RPE, which in turn can facilitate the maintenance of photoreceptor integrity.

The photoreceptors and RPE of human AMD eyes and of DKO *rd8* mice undergo apoptosis likely mediated by the FasL and Fas system as well as the Bcl-2/Bax system (Dunaief et al., 2002; Ding et al., 2009; Wang et al., 2012). We have reported an elevated FasL-mediated RPE apoptosis under inflammation and oxidative stress in the cultured RPE cells of DKO *rd8* mouse than that of WT (Wang et al., 2012). Higher levels of Bax transcript and lower ratios of Bcl2/Bax were also detected in DKO *rd8* mouse eyes compared with WT (Cao et al., 2010). Interestingly, in DKO *rd8* eyes, PEDF increases ONL thickness and decreases apoptotic retinal cells with a decrease in FasL protein, indicating that PEDF might be effective to reverse FasL-mediated apoptosis in DKO *rd8* retina. We detect that no significant decrease of *Fasl* and *Fas* transcripts in the DKO *rd8* retinas with PEDF treatment. This result could be due to the discordant expression of transcript and protein of FasL and Fas in DKO *rd8* mouse eyes. In our previous study, we found higher FasL and Fas protein yet comparable transcript levels in the DKO retina compared with WT (Cao et al., 2010; Wang et al., 2011). In the present study, PEDF significantly down-regulated pro-apoptotic Bax protein and transcript as well as up-regulated anti-apoptotic Bcl-2 protein in DKO *rd8* retina. These findings are in agreement with the PEDF effects in the retinas of Royal College Surgeons rats and retina progenitors R28 cells in culture in which the anti-apoptotic effect of PEDF results from induction of Bcl-2 expression to prevent nuclear translocation of apoptosis-inducing factor (Murakami et al., 2008). An overexpression of Bcl-2 can lead to prevention of Bax translocation and activation, resulting in lower cell apoptosis (Murphy et al., 2000; Ku et al., 2011). Thus, the influence of PEDF on decreasing pro-apoptotic and elevating anti-apoptotic Bcl-2 family members can produce a survival effect in the DKO *rd8* retina.

Another important mechanism of AMD development is the involvement of inflammation (Ding et al., 2009; Xu et al., 2009; Tuo et al., 2012a). Various factors, including smoking, oxidative stress, complement components and inflammatory mediators contribute to parainflammation in human AMD (Xu et al., 2009; Rutar et al., 2011; Wang et al., 2011; Ambati and Fowler, 2012; Colak et al., 2012; Kauppinen et al., 2012; Tarallo et al., 2012). Retinal changes in DKO *rd8* mice are indicative of a chronic inflammatory process, immune dysfunction and activation of innate immunity (Ross et al., 2008; Chu et al., 2012; Tuo et al., 2012b; Ramkumar et al., 2013). Various pro-inflammatory cytokines and related molecules have also been reported in DKO *rd8* retinas and RPE.

As a multifactorial disease, retinal neurons are exposed to the pro-inflammatory cytokines in AMD. Among them, pro-inflammatory cytokines such as TNFα and IL-1β are considered as one of the primary components responsible for the inflammatory response in AMD. Recently, several reports have showed the involvement of IL-17 in the inflammatory pathogenesis of AMD (Liu et al., 2011; Wei et al., 2012; Hasegawa et al., 2013). Moreover, oxidative stress plays a role in the rd in AMD. By using the DKO *rd8* mouse model, our previous studies demonstrated lower transcript expression of *Tnfα* (Tuo et al., 2009; Shen et al., 2011; Tuo et al., 2012b; Ramkumar et al., 2013), *IL1β* (Shen et al., 2011), *IL17a* (Tuo et al., 2012b) and *iNos* (Ramkumar et al., 2013) in mouse retina when treated with anti-inflammatory and/or neuroprotective agents. Thus, we chose these cytokines to detect whether PEDF has anti-inflammatory effects in this mouse model. TNFα, a pro-inflammatory cytokine, is produced by lymphocytes and macrophages, and plays a role in induction of apoptosis. IL-1β, also a pro-inflammatory cytokine, is produced mainly by macrophages and involved in cell proliferation and apoptosis. iNOS, the inducible NOS plays a pivotal role in sustained and elevated NO release. iNOS is released under pathological conditions by inflammation and certain cytokines, including TNFα and IL-1β. Elevated serum IL-17A, a Th17 family cytokine, is reported in AMD patients (Liu et al., 2011). Recently we have shown that IL-17a signal blockade effectively arrested photoreceptor and RPE degeneration in DKO *rd8* mice (Ardeljan et al., ARVO abstr. #1713, 2013). Consequently, all these pro-inflammatory molecules can interact with each other to evoke apoptosis and tissue damage, resulting in rd. The current study demonstrates that PEDF treatment counteracts inflammatory state in neuroretina by down-regulating the inflammatory mediators *Tnfα*, *Il1β*, *iNos* and *Il17a* transcripts to ultimately ameliorate retinal damage and contribute to fewer retinal degenerative lesions in DKO *rd8* mouse retina.

PEDF also decreases the levels of VEGF, a pro-inflammatory molecule that plays a well-recognized role in neovascularization (Reinders et al., 2003). Although VEGF expression is regulated largely by hypoxia and mainly involved with CNV in neovascular AMD, VEGF/PEDF ratio favoring angiogenesis may promote drusen accumulation and trigger progression towards CNV in smoker patients with dry AMD (Pons and Marin-Castano, 2011). Pons et al. also detected increased VEGF levels in RPE/choroids from mice under oxidative stress. An increased VEGF level is documented in DKO *rd8* mice (Herzlich et al., 2009; Tuo et al., 2012c; Ramkumar et al., 2013). Thus, the reduced VEGF expression also indicated that inflammatory and oxidative milieu of PEDF-treated DKO *rd8* eyes is lessened.

In summary, our data show that PEDF stabilizes focal photoreceptor degeneration in DKO *rd8* mice. The protective mechanisms deduced from our data are via anti-apoptotic, anti-inflammatory and anti-angiogenic pathways. The neuroprotective effect of PEDF constitutes a novel approach for potential treatment of AMD.
